# Attitudes and Experiences of Patients Regarding Gender-Specific Aspects of Pain Management

**DOI:** 10.3390/pharmacy12060175

**Published:** 2024-11-22

**Authors:** Carolin Alexandra Boldt, Dirk Keiner, Norman Best, Thilo Bertsche

**Affiliations:** 1 Pharmacy Department, Sophien- und Hufeland-Klinikum gGmbH Weimar, 99425 Weimar, Germanyd.keiner@klinikum-weimar.de (D.K.); 2 Clinical Pharmacy Department, Institute of Pharmacy, Medical Faculty, Leipzig University, 04109 Leipzig, Germany; 3 Drug Safety Center, Leipzig University and Leipzig University Hospital, 04103 Leipzig, Germany; 4 Center for Physical and Rehabilitation Medicine (ZPRM), Sophien- und Hufeland-Klinikum Weimar, 99425 Weimar, Germany; n.best@klinikum-weimar.de

**Keywords:** gender role, pain management, surveys and questionnaires, patient interview, patient chart review

## Abstract

Background: Biological, pharmacological, and socio-cultural aspects influence gender-specific effects in pain management. Methods: Gender-specific aspects of pain management were assessed in a rural outpatient center via semi-structured patient interview: (i) general gender aspects (total population) from 1 = “fully disagree” to 5 = “fully agree”; and (ii) individual pain (matched pairs) via numeric analog scale (NAS) from 0 = “no pain” to 10 = “maximum pain”. Patient charts were assessed for pain management (WHO-ladder). Results: In total, 113 patients were enrolled (59.18 [SD: 12.76] years, 46% female, 54% male, 0% diverse), and 42 were matched into female-male pairs. (i) Women and men agreed that men and women should be treated equally despite biological differences (median: 5 [women] vs. 5 [men]; *p* = 0.789). As a reason for gender-specific aspects, “medication concentration” was reported more frequently by women (*p* = 0.038) and “no answer” by men (*p* = 0.014). (ii) Mean value (SD) of pain (NAS) was 4.0 (SD 2.3) for women and 3.3 (SD 2.6) for men (*p* = 0.215) with a positive correlation between pain management escalation (WHO-ladder) and the pain score (NAS) only in men (r = 0.704, *p* = 0.001). Women rather reported an influence of adverse drug reactions on treatment contentment than men (*p* = 0.042). Conclusions: Although patients pleaded for gender-independent equal treatment, gender-specific differences in pain therapy were found.

## 1. Introduction

Gender-specific aspects in medicine and pharmacy are increasingly coming into clinical focus [1,2]. This applies to both inpatient and outpatient care, and above all to interfaces of care. Pharmacodynamic and, above all, pharmacokinetic factors can cause gender-specific differences in the efficacy and in adverse drug reactions (ADR) [3]. However, the potential gender-specific efficacy of drugs in patients is frequently already due to preclinical development [4]. Drugs, which were tested only on male test animals during preclinical development, may not work as well in women later on or may be more likely to cause ADR in women. Women should be, as a logical consequence, increasingly included in clinical trials. However, women are still underrepresented in most clinical studies, as reported at least from cardiology [5]. In addition to biological and pharmacological aspects, socio-cultural and healthcare parameters may also play a role in gender-specific effects [6]. For instance, participation in colorectal cancer screening programs is higher in women than in men in all age groups [7]. Such differences in the utilization of services in the healthcare system can ultimately have considerable consequences for the entire healthcare system. This is the case, for example, with follow-up costs. The equal treatment of all genders (i.e., more socio-cultural, equity/inequity) is often countered by the desire for individual adaptation to gender-specific factors (i.e., more biological, sameness/differences) [8]. Gender-specific communication differences can also play a decisive role. What is more, the social gender can differ from the genetic sex and queer or transgender people have so far hardly been the subject of gender studies [9]. There is a need to explore new methods for assessing the multiple, nuanced, and intersectional dimensions of gender in pharmacoepidemiology research, such as medication adherence [10].

Pain management is a particularly interesting area in which all these mentioned gender-associated and additional other factors come together [11]. Gender-specific differences in pain sensitivity have been described: Women tend to be more sensitive to various pain stimuli [12]. Such experimental studies indicate differences in pain tolerance between women and men. What is more, clinical studies emphasize that women experience more pain in various diseases, especially in inflammatory processes [13]. Female gender is a predictor for more severe pain after orthopedic surgery with higher intensity and frequency [14]. This could have clinically relevant implications for early mobilization and the development of chronic pain [14]. Various explanations for gender-specific differences in pain management have been given [15]. They range from experience-based and socio-cultural differences between men and women to hormonally and genetically driven sex differences in brain neurochemistry [15]. It has been reported that socio-culturally defined identity factors have a significant influence on the experience, management, and treatment of chronic pain [16]. Unfortunately, such socio-cultural factors have so far been insufficiently investigated in pain management [16]. In addition, those factors can vary greatly, e.g., depending on rural or urban settings or healthcare conditions in different geographical regions with different patient experiences and attitudes, and are also subject to change over time.

Estrogens and gestagens have both pro- and antinociceptive effects due to their receptor distribution in the peripheral and central nervous system. In contrast, androgens have a more antinociceptive effect [17]. Differences in oral bioavailability seem to be particularly important for gender differences [18]. These are caused by different activities of important metabolic enzymes in the intestine and liver [18]. Distribution differences also seem to play a role but are at least partly due to fundamental weight differences between women and men. Pharmacokinetic differences, on the one hand, frequently and sometimes clinically relevantly contribute to gender-specific differences [18]. Those are mainly related to the gender-specific expression of metabolic enzyme systems, such as CYP3A4 and CYP1A2 [18]. Pharmacodynamic differences, on the other hand, appear to be mostly due to modulation by sex hormones [18]. Because of such factors, patients with pain therapy are considered a particularly vulnerable group to gender-dependent effects [18].

Overall, the literature certainly points to a biological influence on gender-specific aspects. However, socio-cultural aspects and health-care parameters appear to have an additional significant influence on those conditions. Therefore, the patient perspective, i.e., their attitudes and experiences, deserves greater consideration in terms of gender-specific aspects. In this regard, not only the university environment but also a rural setting with an interface between outpatient and inpatient care should be examined. This is because differences in gender-specific aspects have been reported depending on whether the patient lives in an urban or rural area [19]. However, data are scarce, particularly in the area of pain therapy.

In this study, therefore, outpatients treated in a rural outpatient center were invited to participate. In addition, the results of a semi-structured patient interview were compared with a review of patient charts. A semi-structured interview was chosen, which allows for standardization on the one hand and a certain flexibility on the other in order to adequately represent such an individual experience as pain. The aim was to investigate patient attitudes and experiences of gender-specific aspects of pain management.

## 2. Materials and Methods

*Participants and setting:* The study was performed in a rural outpatient center of a hospital with a regional-intermediate care mandate and a maximum of 615 beds. Outpatients from the Center for Physical and Rehabilitation Medicine (ZPRM), Sophien- und Hufeland-Klinikum of this hospital were invited to voluntarily participate in a semi-structured interview, followed by a patient chart review. The center provides inpatient and outpatient care under a physician-led interprofessional team of occupational therapists, physiotherapists, and sports therapists. The focus is on outpatient rehabilitation for musculoskeletal disorders, following conservative or surgical treatment. Referrals are made after acute injuries, such as after a slipped disk, after planned surgical procedures, such as the use of prostheses, and in the case of long-term pain and functional limitations in the musculoskeletal system. The general practitioner (GP) or a specialized primary care physician can make the referral.

*Study design:* A prospective semi-structured patient interview was performed. Additional data on pain management was obtained by reviewing the patient chart. The patients were enrolled in the study over a total period of twelve weeks (25 January to 22 February 2024 and 11 March to 24 April 2024). The patient interview was always conducted by one and the same person. The time frame was determined by the availability of the person conducting the interview. The study team had no influence on the patients treated in the outpatient center. The study was divided into the following aims and subjects:

- General gender aspects in the total population
  o Gender-specific effects in pain management;
  o Gender-specific in healthcare communication about pain management.
- Individual pain aspects in matched pairs
  o Pain intensity (compared to pain management according to the WHO-ladder);
  o Contentment with pain management;
  o Adverse drug reactions from pain management.

*Inclusion criteria:* Participation in the study was conditional on the patient being of legal age (i.e., 18 years). In addition, at least one analgesic had to have been taken (at least as an on-demand medication). Exclusion from the study occurred if the medical conditions were not met or if the patient did not give written informed consent. Patients were asked for their written informed consent when they registered so that they could read it in advance during the waiting time. Verbal and written information was provided and written informed consent was obtained before the beginning of the semi-structured patient interview.

*Matched pairs:* The focus of the evaluation in the matched pairs was on the patients’ own subjective, direct experience of pain, including objective parameters from a chart review. In order to be able to directly compare women and men in pairs with similar pain management requirements, one woman and one man were matched in pairs according to their age, body mass index (BMI), and diagnoses as follows:

1. Matching by age group;
2. Matching by body mass index (BMI);
3. Matching by diagnoses (based on the “International Classification of Diseases”: ICD diagnoses) including the severity of the surgical procedure or the course of injury;
4. In case of no match inside the defined groups; Age ±15 years and/or BMI ±5;
5. In case of selection options, consideration of comorbidities as similar as possible.

Non-matched patients were excluded from the matched pairs analysis.

*Patient interview:* As a basis for the semi-structured interview, the author team designed a questionnaire. For statements in closed questions, “I-statements” were used with answer options on a 5-point Likert scale:

- 1 (“I strongly disagree”);
- 2 (“I rather disagree);
- 3 (“I partly disagree and partly agree”);
- 4 (“I rather agree”);
- 5 (“I fully agree”).

Questions included the option “no answer” as well as the option “other”.

*Current pain situation:* Patients marked on a written visual analog scale (VAS) the value that seemed most applicable to them from “no pain” to “maximum imaginable pain”. The exact numerical analog value (NAS) on the scale was then measured and given as a value of the NAS.

*Design of the questionnaire as the basis for the interview*: The following published questionnaires were considered:

- “Revised American Pain Society Patient Outcome Questionnaire” [APS-POQ-R] [20];
- ADR in the APS-POQ-R was supplemented according to the Medication Side-Effects Checklist [MSEC] [21,22];
- Communication about ADR [23];
- The validated Treatment Satisfaction Questionnaire (TSQM) was used to assess satisfaction with the pain management received and relevant questions were adapted specifically for pain management [24];
- The validated questionnaire “Gender Awareness in Medicine Scale” (N-GAMS), which comprises three sub-areas: gender sensitivity, and gender role in relation to patients and physicians [25].

The questions were selected and tailored to the environment and objectives. Additional questions and aspects (e.g., about pharmacies) were added. Before the questionnaire was applied to patients in this study, it was pre-tested independently of the study participants. The test subjects were medical laypersons as well as physicians and pharmacists with experience in the field of gender and pain management.

*Statistics and data evaluation:* Descriptive data collection and analysis were carried out using Excel (Microsoft for Windows 11) and PSPP 2.0.1 (GNU Project Free statistical software, GNU General Public License, 21 March 2024). Relative and absolute frequencies, as well as the median, 25% and 75% quartiles or standard deviation (SD), were chosen as appropriate. Relationships and correlations were examined using the Chi-square function and Spearman correlation, as well as Kendall’s tau-b. Gender differences in the total population were analyzed using the Mann–Whitney test with the assumption of an independent, ordinal sample. To test for significant differences between men and women in matched pairs, a paired sample with ordinal data was assumed and the Wilcoxon test was performed. For the interval-scaled NAS, a paired *t*-test was performed. In addition, central tendencies of the mean values were examined using an analysis of variance according to the corresponding test (Kruskal–Wallis, post hoc test with Bonferroni correction).

## 3. Results

### 3.1. Patient Characteristics

For the overall population analysis, 113 patients were enrolled. The average age of those patients was 59.18 years with a standard deviation (SD) of 12.76 years. Overall, 46% (N = 52) of respondents named themselves female, 54% (N = 61) male, and 0.0% (N = 0) diverse.

For the matched pairs analysis, 84 of those 113 patients were matched in pairs of 42 women and 42 men with comparable age, body mass index (BMI), and diagnoses (ICD 10 code) as follows: fracture of the femur (ICD 10 code: S72): 2.4% (N = 2); fracture of the lower leg, including the upper ankle joint (S82): 4.8% (N = 4); rotator cuff lesion (M75), shoulder joint injury/disease (M24-25), and fracture of shoulder and humerus bone (S42): 21% (N = 18); hip total endoprosthesis (M16; Z96.64): 19% (N = 16); spinal problems (M40–M54): 19% (N = 16); and knee total endoprosthesis (M17; Z96.65): 33% (N = 28). The average age of those patients in matched pairs was 60.19 (SD 10.61) years for women and 59.93 (SD 12.45) years for men.

### 3.2. General Gender Aspects in the Total Population

#### 3.2.1. Gender-Specific Effects in Pain Management

As shown in Table 1, on a Likert-scale ranging from “1 = I strongly disagree” to “5 = I strongly agree”, the following ratings were given in median: Women and men strongly disagreed that women and men were treated differently in pain management (median: 1 [women] vs. 1 [men]; *p* = 0.497). Women and men strongly agreed that men and women should be treated equally, despite biological differences (median: 5 [women] vs. 5 [men]; *p* = 0.789). Women and men rather agreed that gender-specific effects of medication exist (median: 4 [women] vs. 4 [men]; *p* = 0.546). Women and men rather or partly disagreed and partly agreed that they would like to obtain more information on gender-specific effects (median: 3 [women] vs. 2 [men]; *p* = 0.191). The correlation analysis showed that patients who believed in a gender-specific effect of pain medication would also like to receive more information on this topic (Spearman-Rho correlation r(s) = 0.55, *p* < 0.001, N = 91, Kendall tau b value τ = 0.46, *p* < 0.001).

**Figure 1:**
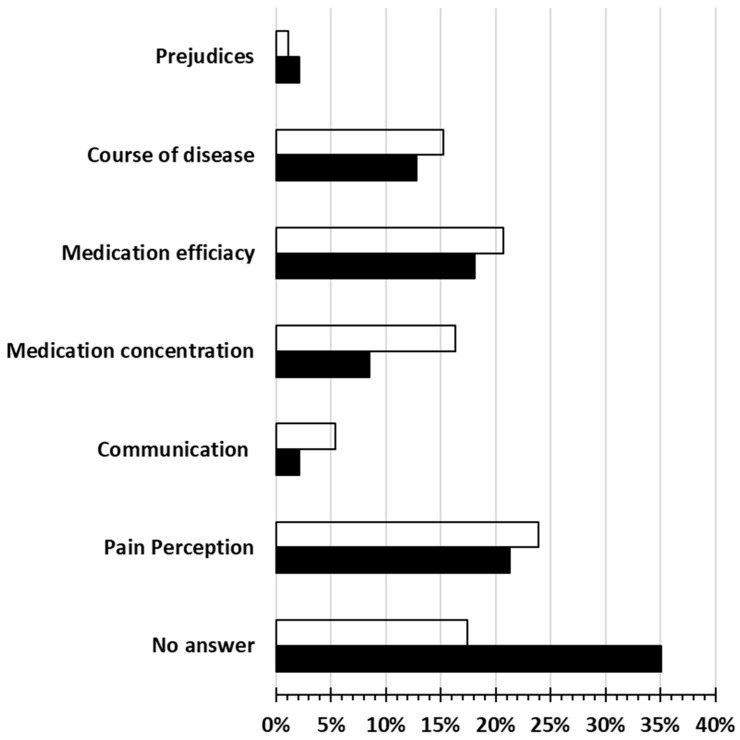
General gender aspects in the total population. Gender-specific effects in pain management. Response frequencies in given options to the question “What could be the reasons for the use of different therapies in men and women?”. Chi-square test for the proportion of “yes” and “no” as an important aspect. Multiple answers were possible. Assessed in the overall population of 113 participating patients. Transparent bars: women; filled bars: men. Significant differences between women and men: “Medication concentration” (*p* = 0.038) and “no answer” (*p* = 0.014).

As presented in Figure 1, except from “no answer” to the question “What could be the reasons for the use of different pain therapies in women and men?”, “pain perception” and “medication efficacy” were reported from both women and men to be the two main possible causes for diversities. A significant preference of women was revealed for “medication concentration” (*p* = 0.038) and a preference in favor of men for “no answer” (*p* = 0.014).

#### 3.2.2. Gender-Specific in Healthcare Communication About Pain Management

As presented in Table 2, without any significant differences between women and men, women and men fully disagreed (median: 1 [women, men]) that gender-specific differences in communication with their physicians exist (*p* = 0.674). They fully disagreed that they had been informed about gender-specific effects of medication by their physician (*p* = 0.898) and at their pharmacy (*p* = 0.889), as well as that they were not taken seriously because of their gender by their physician (*p* = 0.076) and at their pharmacy (*p* = 0.621).

### 3.3. Individual Pain Aspects in Matched Pairs

#### 3.3.1. Pain Intensity

In a matched paired analysis, the mean value (SD) of pain according to the numeric analog scale (NAS) reaching from 0 to 10 was not significantly different comparing women with an NAS of 4.0 (SD 2.3) and men with an NAS of 3.3 (SD 2.6; *p* = 0.215). A positive correlation was found between pain management according to the WHO-ladder and the pain score according to NAS in men (r = 0.704, *p* = 0.001), but not in women (r = 0.028, *p* = 0.862).

#### 3.3.2. Contentment with Pain Management

As presented in Table 3, both, women and men (median: 5 [women, men]; *p* = 0.787) fully agreed that they were content with their pain relief. In median, women fully (median: 5) and men partially agreed (median: 4) that they are generally content with their pain management (*p* = 0.307). Women and men partly disagreed and partly agreed (median: 3 [women, men]) to be involved in decisions about their treatment (*p* = 0.772). They fully agreed (median: 5 [women, men]) to be content with the communication with their physician (*p* = 0.741) and at their pharmacy (*p* = 0.430). They agreed fully (median: 5 [women]) or rather (median: 4 [men]) to have raised ADR and drug-related problems with their physician (*p* = 0.477), and rather (median: 2 [women]) or fully (median: 1 [men]) disagreed to have raised adverse drug reactions (ADR) and drug-related problems in their pharmacy (*p* = 0.594).

#### 3.3.3. Adverse Drug Reactions (ADR) from Pain Management

As presented in Table 4, women and men (median: 1 [women, men], *p* = 0.607) fully disagreed to have ADR from their pain management. Women partly disagreed and partly agreed (median: 3) that ADR of pain management is a burden while men rather disagreed (median: 2, *p* = 0.104). Women and men rather disagreed (median: 2 [women, men]) that they had stopped taking their pain management since the ADR was too much of a burden (*p* = 0.177). A significant difference was found when comparing women and men assessing the ADR influence on their treatment contentment: women (median: 3) partly disagreed and partly agreed while men (median: 1) fully disagreed (*p* = 0.042).

## 4. Discussion

Although we explicitly asked about non-binary orientations and would have liked to analyze them in the sense of an inclusive approach, only male and female perspectives were mentioned in the self-assessment. Unfortunately, this means that the diverse aspects of non-binary or transgender persons cannot be analyzed within the scope of this study. Furthermore, it is not possible to answer whether those persons were not represented at all or simply did not admit to it.

Regardless of their gender, patients in this study agreed that they received effective pain management. Women and men mainly fully disagreed with having ADR from their pain management. Gender-specific aspects in routine pain management currently play a relatively minor role, independently from gender. Patients reported only limited interest in further information on this subject from the prescribing physician or at the dispensing pharmacy. Women and men agreed that biological gender-specific effects in pain management exist. However, patients did not see the need to take them into account in routine care. They started to receive hardly any information about gender-specific effects from their physicians and at their pharmacy. Apart from all those gender-independent results, additionally, some significant gender-specific differences were found: Firstly, different medication concentrations and thus pharmacokinetic differences were mentioned by women more frequently than by men as relevant factors for gender-specific effects in pain management, while men were significantly more often unwilling or unable to answer this question. Secondly, only for men, a significant correlation between pain intensity with the WHO ladder was found. Thirdly, the ADR influence on treatment satisfaction showed significantly higher agreement among women than men. Fourth, it was also apparent that patients who believe in a gender-specific effect of pain management would also like to receive more information on this topic from their healthcare providers significantly more frequently.

There are numerous indications that pain management for women is more complex than for men. This is due to biological as well as socio-cultural aspects. Ultimately, women may also suffer more from pain in general [12]. This explains why the association between escalated pain management (according to the WHO-ladder) and escalated pain intensity (according to NAS) in the study only showed a significant correlation in men. This could be an indication of inadequate provision of pain management for women. An additional indication for this hypothesis could be that—even if not significant—a purely numerical comparison of the matched pairs showed a higher pain intensity in women compared to the corresponding men with comparable age, BMI, and diagnoses. This once again underlines the literature findings that the pain situation in women could be more severe and is, therefore, more difficult to manage via appropriate pain management than in men. Even an age-dependent influence of gender-specific aspects is under discussion [26] which underlines the meaningfulness of the age reference in the matched pairs formation.

The preferential inclusion of male animals in preclinical development and the subsequent preference for male probands and patients in clinical trials may result in suboptimal efficacy of approved drugs and a higher incidence of ADRs [27]. This goes in line with the results of the study presented here that women are more likely to state that an ADR can influence their treatment satisfaction. This may also speak for an increased incidence of burdensome ADR with anti-inflammatory drugs in women [28]. This is logically related to the fact that preclinical and clinical drug development has so far frequently not been directed at women and, therefore, ADR may have already been experienced more frequently in women than in men. Not only do genetic factors influence gender-specific pain sensitivity, but pain-coping behavior has also been described as different in the current literature [29]. Such coping strategies can be techniques for patients to cope with pain. They involve changing dysfunctional pain-associated cognitions, emotions, and behaviors that contribute to triggering or worsening pain. Different coping strategies can also explain different ways of dealing with ADR, as found in the current investigation presented here.

It is known that there are differences between women and men when communicating about pain [25]: Women tend to exhibit more detailed and factual language. As also described in [3], men use fewer words and focus on the sensory aspects of pain. In the study presented here, communication with the physician or at the pharmacy revealed no gender-specific differences or prejudices. Women and men were satisfied with their current pain management prescribed by their physicians. Regardless of gender, however, they felt moderately involved in decision-making about their pain management. This is in contrast to the fact that both felt that communication with the physician and at the pharmacy was very satisfactory. ADR was discussed quite well with the physicians of women and men. Both genders agreed that the pharmacy is less of a contact for ADR. Not surprisingly, patients who believe in a gender-specific effect of pain management would also like to receive information on this topic significantly more frequently. The aim of a particularly interesting review [29] was to examine gender-specific norms in relation to men and women with pain and gender bias in the treatment of pain. Theoretical concepts of hegemonic masculinity and andronormativity were also to be considered. Such indications were also found in this study; for example, when men complained of less pain, they were possibly treated more adequately, perceived less influence of ADR on their pain management, and reported fewer reasons for gender differences than women.

We deliberately chose a rural setting in this study since we suspected that the setting could have an influence on the perception of gender-dependent effects. However, we have not yet been able to demonstrate in this study that a rural setting would have yielded different results, e.g., a significantly larger number of non-binary individuals. This should, therefore, be the subject of future comparative studies in urban settings. Conclusions can then be drawn from this comparison as to whether tailor-made measures are necessary, particularly in rural areas. The generalizability of the study in its current form is limited by the fact that it was performed in a single center in a rural setting. Future research could include a more diverse sample in terms of ethnicity, socioeconomic status, or geographical regions to capture different patient experiences and attitudes. This could probably also be achieved by including an urban center.

Concrete conclusions for the optimization of patient care with a focus on pain therapy can also be derived from the results of this study to date. Firstly, awareness of gender-specific effects in pain therapy should be increased while educating healthcare providers. In the next step, this group can act as a multiplier in order to successively improve the gender-related knowledge of their patients. Ultimately, evidence-based gender-specific aspects should also be better taken into account in clinical guidelines.

The results show that there are either no non-binary personalities in our survey group or that the people concerned do not want to come out even in an anonymous survey. In general, the respondents are skeptical about gender differences. In principle, they accept biological differences, but do not see any organizational gender-specific differences in treatment in their treatment routine. Surprisingly, however, we indeed did find gender-dependent differences in terms of a better desirable connection between the WHO gradual classification and pain intensity in men. Women, on the other hand, feel more strongly influenced by ADR than men. Those who are already open to gender-specific differences are also open to further information. The latter fact, in particular, makes it difficult to reach people who find it difficult to access training offers and information.

In conclusion, the article makes an important contribution to the field by highlighting gender differences in pain management, including patient perspectives. To open the door for future pharmacoepidemiological research, a more in-depth examination of the different gender identities, addressing socio-cultural factors in more detail, and developing additional clinical applications can further develop and build on the existing findings from this study.

### Limitations

The following limitations should be considered while interpreting the results:

(i) The study was only conducted in one hospital. Deliberately, a smaller rural outpatient center was chosen as previous data on similar topics tended to come from large university centers. Nevertheless, the results should not be generalized uncritically;
(ii) The questionnaire that served as the basis for the interview was newly developed for the questions and tested in advance. Although this questionnaire was based on validated instruments, the newly compiled questionnaire was not validated again as a whole with regard to the clinical outcomes. so one-to-one derivations for direct clinical relevance should be treated with caution and the measurement accuracy is limited in this respect;
(iii) Although the semi-structured approach makes it possible to respond individually to the patient, personal influencing factors could not be completely excluded in the individual interview. However, all interviews were conducted by the same person in order to exclude the influence of different people during the interview;
(iv) The patients’ assessments depend on their current life situation and their age. They are, therefore, in flux and should be the subject of further re-evaluation.

## 5. Conclusions

The enrolled patients of a rural outpatient center agreed to receive an effective pain treatment, regardless of their gender. In most areas, patients did not perceive any gender-specific differences. What is more, they did not consider them to be necessary to be considered in routine pain management. However, individual indicators, such as the correlation between pain intensity and pain management, the influence of stressful ADR on pain management, and pharmacokinetics, showed gender-dependent differences.

In our opinion, the concerning results of this study are not only statistically significant but also clinically relevant. They show that gender aspects are frequently not in the consciousness of patients (and physicians) but that actual differences can be observed. These are often not only due to biological differences but also extend to gender-specific differences in treatment, which can lead to inappropriate care depending on gender, and they should not be tolerated. Often, such gender-dependent treatment differences are not even deliberately caused, as evidenced by the lack of awareness. This is underlined by the results obtained in this study.

This way, the results presented here show that awareness of gender-specific aspects of pain management should be increased in the future.

## Figures and Tables

**Table 1:** General gender aspects in the total population. Gender-specific effects in pain management. Answer scale according to Likert from 1 = I fully disagree, 2 = I rather disagree, 3 = I partly disagree and partly agree, 4 = I rather agree, to 5 = I fully agree. Assessed in the overall population of 113 participating patients. The number of responding patients is displayed.

Question	Women [Median (Q25/Q75); Responding Patients]	Male [Median (Q25/Q75); Responding Patients]	*p*-Value [Women vs. Men]
I believe that men and women are treated differently in pain management.	1 (1/2); n = 44	1 (1/2.5); n = 51	0.497
I believe that men and women should be treated equally in of pain management, despite biological differences.	5 (3/5); n = 44	5 (3.5/5); n = 51	0.789
I believe there are gender-specific effects of pain management.	4 (3/5); n = 42	4 (2/4); n = 49	0.546
I would like to get more information on gender-specific effects in pain management.	3 (1/4); n = 42	2 (1/4); n = 49	0.191

**Table 2:** General gender aspects in the total population. Gender-specific in health-care communication about pain management. Answer scale according to Likert from 1 = I fully disagree, 2 = I rather disagree, 3 = I partly disagree and partly agree, 4 = I rather agree, to 5 = I fully agree. Assessed in the overall population of 113 participating patients. The number of responding patients is displayed.

Question	Women [Median, (Q25/Q75); Responding Patients]	Men [Median, (Q25/Q75); Responding Patients]	*p*-Value [Women vs. Men]
There are gender-specific differences in communication with physicians.	1 (1/1); n = 43	1 (1/2); n = 52	0.674
I have been informed about the gender-specific effects of medication by the physician.	1 (1/1); n = 47	1 (1/1); n = 59	0.898
I have been informed about the gender-specific effects of medication in the pharmacy.	1 (1/1); n = 42	1 (1/2); n = 44	0.899
I was not taken seriously by the physician because of my gender.	1 (1/1); n = 51	1 (1/1); n = 60	0.076
I was not taken seriously in the pharmacy because of my gender.	1 (1/1); n = 48	1 (1/1); n = 48	0.621

**Table 3:** Individual pain aspects in matched pairs. Contentment with pain management. Answer scale according to Likert from 1 = I fully disagree, 2 = I rather disagree, 3 = I partly disagree and partly agree, 4 = I rather agree, to 5 = I fully agree. Assessed in the matched pairs of corresponding 42 women and 42 men of the 113 participating patients. The number of responding patients is displayed. ADR: adverse drug reactions.

Question	Women [Median, (Q25/Q75); Responding Patients]	Men [Median, (Q25/Q75); Responding Patients]	*p*-Value [Women vs. Men]
I am content with the pain relief.	5 (4/5); n = 42	5 (3/5); n = 42	0.787
I am generally content with my pain management.	5 (4/5); n = 42	4 (3/5); n = 41	0.307
I was involved in decisions about my treatment.	3 (1/5); n = 41	3 (2/5); n = 40	0.772
I am content with the communication with my physician.	5 (5/5); n = 35	5 (4/5); n = 35	0.741
I am content with the communication in my pharmacy.	5 (5/5); n = 30	5 (5/5); n = 27	0.430
I have raised ADR and drug-related problems with my physician.	5 (3/5); n = 26	4 (2/5); n = 23	0.477
I have raised ADR and drug-related problems in my pharmacy.	2 (1/4); n = 15	1 (1/3); n = 16	0.594

**Table 4:** Individual pain aspects in matched pairs. Adverse drug reactions (ADR) from pain management. Answer scale according to Likert from 1 = I fully disagree, 2 = I rather disagree, 3 = I partly disagree and partly agree, 4 = I rather agree, to 5 = I fully agree. Assessed in the matched pairs of corresponding 42 women and 42 men of the 113 participating patients. The number of responding patients is displayed. ADR: adverse drug reactions.

Question	Women [Median, (Q25/Q75); Responding Patients]	Men [Median, (Q25/Q75); Responding Patients]	*p*-Value [Women vs. Men]
I have ADR from my pain management.	1 (1/1); n = 42	1 (1/1); n = 42	0.607
The ADR of pain management are a burden.	3 (2/4); n = 21	2 (1/4); n = 17	0.104
I have stopped taking my pain management since the ADR were too much of a burden.	2 (1/4); n = 22	2 (1/3); n = 16	0.177
The ADR influence my treatment contentment.	3 (1/4); n = 22	1 (1/3); n = 17	0.042

## Data Availability

The data presented in this study are available on request from the corresponding author, insofar as this is justifiable for ethical and data protection reasons.
